# Predictors of unplanned hospital readmissions and emergency department revisits in patients with acute ischemic stroke

**DOI:** 10.3389/fneur.2025.1683753

**Published:** 2025-10-21

**Authors:** Ammar Jumah, Jennifer Ro, Tianwen Ma, Javacia Owens, Yunong Wu, Danielle Starnes, Hannah Christopher, Debra Blanke, Laura Henriquez, Samir R. Belagaje, Nino Kvantaliani, Jacquelyn Cabral, Teri Walczak, Judy Collier, Kourtni Mack, Fadi Nahab

**Affiliations:** ^1^Department of Neurology, Emory University, Atlanta, GA, United States; ^2^Department of Biostatistics and Bioinformatics, Rollins School of Public Health, Emory University, Atlanta, GA, United States

**Keywords:** unplanned hospital revisits, emergency department revisits, acute ischemic stroke, telemedicine, prevention

## Abstract

**Objectives:**

We aim to identify factors associated with emergency department (ED) revisits and hospital readmissions after acute ischemic stroke (AIS) diagnosis and to determine if early outpatient follow-up can reduce readmissions.

**Methods:**

We retrospectively identified all AIS patients discharged from a hospital network, from October 1, 2022 to March 31, 2024. Baseline characteristics, inpatient metrics and post-discharge outpatient follow-up were assessed to identify factors associated with ED revisits and readmissions to the healthcare system within 90-days.

**Results:**

Of 1,973 patients, 464 (23.5%) had ED visits within 90 days and 263 (13.3%) had hospital readmission within 90 days. The median age was 68 [IQR 58, 77]. In multiple logistic regression analyses, factors independently associated with 90-day ED visit were history of heart failure (HF) (OR 1.46, 95% CI 1.11–1.93; *p* = 0.007), diabetes mellitus (DM) (OR 1.41, 95% CI 1.12–1.77; *p* = 0.003), atrial fibrillation (AF) (OR 1.47, 95% CI 1.13–1.92, *p* = 0.004) and an increasing Charlson comorbidity index (CCI) score (OR 1.10, 95% CI 1.03–1.18), *p* = 0.003). Factors associated with 90-day readmission were HF (OR 1.51, 95% CI 1.08–2.11, *p* = 0.015), DM (OR 1.50, 95% CI 1.13–2.01, *p* = 0.006), AF (OR 1.40, 95% CI 1.00–1.94, *p* = 0.047) and increasing CCI score (OR 1.12, 95% CI 1.03–1.21, *p* = 0.006). Discharge to inpatient rehabilitation or skilled nursing facility (vs. home or home health) were associated with 90-day ED revisits and hospital readmissions. Patients who completed early (<30 days) outpatient stroke clinic follow-up had a lower likelihood of 90-day readmission (OR 0.68, 95% CI 0.52–0.90; *p* = 0.006).

**Conclusion:**

Patients with certain comorbidities including HF, DM, AF and those with a higher CCI score have a higher likelihood of a 90-day ED revisit and hospital readmission. Unplanned hospital readmissions may be preventable with early outpatient visits in a dedicated stroke clinic after discharge for AIS patients.

## 1 Introduction

Stroke remains to be a leading cause of death and disability worldwide (1), with outcomes in acute ischemic stroke (AIS) significantly influenced by a myriad of factors (2). These outcomes depend not only on acute patient care, including patient access to stroke diagnosis, treatment, and the rapid identification of treatment-related complications, but also on post-hospitalization care and secondary stroke prevention (3–5). Additionally, outcomes are shaped by patients' demographics (age, sex, etc.), comorbidities, stroke severity, post-discharge disposition, socioeconomic status and may be impacted by hospital revisits as well as early outpatient follow-up where stroke risk factors and etiology continue to be evaluated (3, 6–9).

Stroke is among the leading causes of unplanned hospital readmissions, which negatively impacts both healthcare costs and patient outcomes (10). In one systematic review and meta-analysis, it was shown that the readmission rate in patients with stroke was as high as 17% in the first month, and 42% in the year following the event (3), despite the advancements in the management of AIS. Therefore, limiting the rate of unplanned hospital and emergency department (ED) revisits, as a part of secondary stroke prevention for patients who suffer from AIS is of utmost importance.

The objective of this study was to evaluate the factors most likely associated with 90-day unplanned ED visits and hospital readmissions after being hospitalized for AIS. We also studied the impact of early transitional care outpatient follow-up by a centralized specialty stroke clinic.

## 2 Methods

### 2.1 Study setting and population

This was a retrospective study of all patients admitted primarily for a diagnosis of AIS from October 1st, 2022, to March 31st, 2024 to Emory Healthcare, the largest academic health system in the state of Georgia with locations primarily in metropolitan Atlanta. Data was collected using ICD-10 codes for ischemic strokes and retrieved directly from the electronic medical record. The following inclusion criteria were applied: (1) Adult patient population, who were (2) hospitalized with a diagnosis of AIS. We excluded patients who (1) left against medical advice, (2) were transferred to another acute care facility, (3) discharged to a long-term acute care or hospice or (4) had died prior to their discharge. For patients with multiple discharges, we only considered the earliest one. Readmission and ED revisits were categorized as “All-cause” and captured only those in our academic healthcare system. In our institution, telemedicine is conducted either via phone or video interviews primarily decided on by an agreement between patients and their providers. This study was approved by the institutional review board and the need for informed consent was waived due to the retrospective nature of the study.

### 2.2 Covariates

Baseline characteristics, lab results, inpatient metrics and post-discharge outpatient follow-up were assessed to identify factors associated with ED revisits and hospital readmission within 90 days after hospital discharge within the healthcare system. Early outpatient follow-up was defined as outpatient visits (telehealth or in-person) conducted within 30 days of being discharged from the hospital. Baseline characteristics included age, sex, race, and comorbid conditions were determined using the Charlson comorbidity index (CCI). This index is a scoring system used to predict the mortality risk for patients based on their disease or condition (11–13). A score of zero indicates no comorbidities and as the score increases so does the predicted mortality rate (14). In this study, we also assessed various health conditions, such as diabetes mellitus (DM), heart failure (HF), hypertension (HTN), chronic kidney disease (CKD), hyperlipidemia, obesity, atrial fibrillation (AF) and the National Institutes of Health Stroke Scale (NIHSS) on index admission.

### 2.3 Statistical analysis

A database was created using an Excel spreadsheet to store and analyze the collected data. For continuous covariates, medians and IQR ranges were provided due to the non-normality assumption. For categorical covariates, frequency and proportion values were provided. For univariate comparisons, Wilcoxon rank sum tests and Chi-squared tests were performed on 90-day unplanned hospital and ED revisits for continuous and categorical covariates, respectively. Univariate and multiple logistic regression models were fitted to examine the association between 90-day unplanned hospital and ED revisits and covariates defined in the previous section. For univariate comparisons, if a covariate had missing values, the percentages and *p*-values were computed without missing values. For logistic regression models, a complete-case analysis approach was applied. For the multiple logistic regression model, the initial model included predictors of which *p*-values from univariate comparisons were <0.1. A stepwise model selection with both forward and backward selection was applied. The Akaike Information Criterion (AIC) was used as the selection criterion. Parameter estimates, 95% confidence intervals (CIs), and p-values were reported. All statistical analyses were performed in R, Version 4.3.2. *P*-values <0.05 were considered statistically significant.

## 3 Results

### 3.1 Emergency department revisits

Of 1,973 patients including in the study cohort, 464 (23.5%) had an all-cause ED revisit within 90 days. The median age was 68 [IQR 58, 77], 949 (48.1%) female, 1,146 (60.7%) African American, and 1,921 (97.4%) were from urban areas. History of HF, CKD, ESRD, DM, AF and obesity increased the likelihood of an ED revisit within 90 days in univariate analysis (Table 1). The final logistic regression model suggested that factors significantly associated with 90-day ED visit were history of HF (OR 1.46, 95% CI 1.11–1.93; *p* = 0.007), DM (OR 1.41, 95% CI 1.12–1.77; *p* = 0.003), AF (OR 1.47, 95% CI 1.13–1.92, *p* = 0.004), an increasing CCI score (OR 1.10, 95% CI 1.03–1.18), *p* = 0.003), and discharge to inpatient rehabilitation center (OR 1.74, 95% CI 1.22–2.47, *p* = 0.002) or skilled nursing facility (OR 1.66, 95% CI 1.13–2.45, *p* = 0.01) vs. home or home health (Table 2). Living in a rural (vs. urban) area was independently associated with a lower likelihood of 90-day ED visit (OR 0.29, 95% CI 0.10–0.82).

**Table 1:** Demographics and comorbidities associated with ED revisits within 90 days.

**Variable**	**Levels (categorical)**	**Marginal statistics**	**No (1,509, 76.5%)**	**Yes (464, 23.5%)**	***p*-value**
**Baseline demographic data**					
Age median, [IQR]		68 [58, 77]	68 [57, 76]	69.5 [60, 79]	**<0.001**
Gender *N*, (%)	Female	949 (48.1)	711 (47.1)	238 (51.3)	0.13
	Male	1,024 (51.9)	798 (52.9)	226 (48.7)	
Race and ethnicity *N*, (%)	American Indian, Alaska Native	4 (0.2)	4 (0.3)	0	0.18
	Asian	85 (4.5)	69 (4.8)	16 (3.5)	
	Black	1,146 (60.7)	847 (59.1)	299 (66.0)	
	Hispanic	76 (4.0)	59 (4.1)	17 (3.7)	
	Native Hawaiian, other Pacific Islander	1 (0.1)	1 (0.1)	0	
	Other	20 (1.1)	17 (1.2)	3 (0.7)	
	White	555 (29.41)	437 (30.5)	118 (26.0)	
Rural *N*, (%)		52 (2.6)	48 (3.2)	4 (0.9)	**0.01**
Low-density lipoprotein median, [IQR]		94 [66, 124]	95 [67, 125]	89 [63, 123]	0.15
Length of stay median, [IQR]		4.3 [2.6, 8.1]	4.1 [2.3, 7.5]	5.2, [3.1, 9.1]	**<0.001**
Charlson comorbidity score median, [IQR]		2.0 [0.0, 3.0]	2.0 [0.0, 3.0]	2.0 [2.0, 4.0]	**<0.001**
Stroke education *N*, (%)		1,403 (71.1)	1,064 (70.5)	339 (73.1)	0.32
**Comorbidity** ***N*****, (%)**					
HF		471 (23.9)	302 (20.0)	169 (36.4)	**<0.001**
CKD		545 (27.6)	368 (24.4)	177 (38.1)	**<0.001**
ESRD		63 (3.2)	41 (2.7)	22 (4.7)	**0.044**
Diabetes		883 (44.7)	625 (41.4)	258 (55.6)	**<0.001**
HTN		1,381 (70.0)	1,054 (69.8)	327 (70.5)	0.84
Obesity		376 (19.1)	271 (18.0)	105 (22.6)	**0.03**
AF		397 (20.1)	266 (17.6)	131 (28.2)	**<0.001**
Hyperlipidemia *N*, (%)		1,306 (71.5)	1,008 (72.4)	298 (68.8)	0.17
HbA1c *N*, (%)	<5.7	561 (42.1)	426 (42.1)	135 (41.8)	0.70
	5.7–6.4	360 (27.0)	278 (27.50)	82 (25.4)	
	6.5–6.9	92 (6.9)	71 (7.0)	21 (6.5)	
	>6.9	321 (24.0)	236 (23.3)	85 (26.3)	
NIHSS *N*, (%)	0	473 (24.8)	385 (26.5)	88 (19.5)	**<0.001**
	1–4	854 (44.8)	662 (45.5)	192 (42.5)	
	5–15	452 (23.7)	325 (22.4)	127 (28.1)	
	16–20	68 (3.6)	48 (3.3)	20 (4.4)	
	>20	59 (3.1)	34 (2.3)	25 (5.5)	
Thrombectomy *N*, (%)		108 (5.5)	82 (5.4)	26 (5.6)	0.98
Discharge destination *N*, (%)	Home/home health	1,459 (73.9)	1,158 (76.7)	301 (64.9)	**<0.001**
	Inpatient rehab	290 (14.7)	202 (13.4)	88 (19.0)	
	Skilled nursing facility	224 (11.4)	149 (9.9)	75 (16.2)	

AF, atrial fibrillation; CCI, Charlson comorbidity index; CKD, chronic kidney disease; DM, diabetes mellitus; ESRD, end stage renal disease; HF, heart failure; HTN, hypertension; IQR, interquartile range; NIHSS, national institutes of health stroke scale; OR, odds ratio. Bold values indicate statistical significance.

**Table 2:** Multiple logistic regression after model selection for ED revisits within 90 days.

**Variable**	**OR 95% CI**	***p*-value**
AF	1.47 (1.13–1.92)	**0.004**
CKD	1.26 (0.97–1.62)	0.081
DM	1.41 (1.12–1.77)	**0.003**
HF	1.46 (1.11–1.93)	**0.007**
Increasing CCI	1.10 (1.03–1.18)	**0.003**
Rural areas	0.29 (0.10–0.82)	**0.02**
**Discharge destination**		
Inpatient rehab	1.60 (1.20–2.15)	**0.002**
Skilled nursing facility	1.58 (1.14–2.18)	**0.006**
Home/Home health	Reference	

AF, atrial fibrillation; CCI, Charlson comorbidity index; CKD, chronic kidney disease; DM, diabetes mellitus; HF, heart failure; IQR, interquartile range; OR, odds ratio; N/A, not applicable. Bold values indicate statistical significance.

### 3.2 Hospital readmission

Of 1,973 patients, 263 (13.3%) had 90-day all-cause readmissions to the healthcare system, respectively. Length of stay, increasing age, CCI score, history of HF, CKD, DM, AF, hyperlipidemia, obesity, discharge to inpatient rehabilitation or skilled nursing facility, and NIH stroke scales were associated with hospital re-admission within 90 days in univariable analysis (Table 3). The final multiple logistic regression model suggested that HF (OR 1.51, 95% CI 1.08–2.11, *p* = 0.015), DM (OR 1.50, 95% CI 1.13–2.01, *p* = 0.006), AF (OR 1.40, 95% CI 1.00–1.94, *p* = 0.047), increasing CCI score (OR 1.12, 95% CI 1.03–1.21, *p* = 0.006) and discharge to inpatient rehabilitation center (OR 1.74, 95% CI 1.22–2.47, *p* = 0.002) or skilled nursing facility (OR 1.66, 95% CI 1.13–2.45, *p* = 0.01) were significantly associated with 90-day hospital readmission (Table 4).

**Table 3:** Demographics and comorbidities associated with hospital readmissions within 90 days.

**Variable**	**Levels (categorical)**	**Marginal statistics**	**No (1,710, 86.7%)**	**Yes (263, 13.3%)**	***p*-value**
**Baseline demographic data**					
Age Median, [IQR]		68 [58, 77]	67.5 [57, 77]	71 [61.5, 80]	**<0.001**
Gender *N*, (%)	Female	949 (48.1)	822 (48.1)	127 (48.3)	1.00
	Male	1,024 (51.9)	888 (51.9)	136 (51.7)	
**Race and ethnicity** ***N*****, (%)**	American Indian, Alaska Native	4 (0.2)	4 (0.3)	0 (0.0)	0.97
	Asian	85 (4.5)	75 (4.6)	10 (3.9)	
	Black	1,146 (60.7)	988 (60.6)	158 (61.5)	
	Hispanic	76 (4.0)	65 (4.0)	11 (4.3)	
	Native Hawaiian, Other Pacific Islander	1 (0.1)	1 (0.1)	0 (0.0)	
	Other	20 (1.1)	18 (1.1)	2 (0.8)	
	White	555 (29.4)	479 (29.4)	76 (29.6)	
Rural *N*, (%)		52 (2.6)	49 (2.9)	3 (1.1)	0.16
Low-density lipoprotein Median, [IQR]		94 [66, 124]	95 [67, 124]	86 [60.8, 128.3]	0.087
Length of stay Median, [IQR]		4.3 [2.6, 8.1]	4.2 [2.4, 7.8]	5.3 [3.1, 9.9]	**0.001**
Charlson comorbidity score Median [IQR]		2.0 [0.0, 3.0]	2.0 [0.0, 3.0]	3.0 [2.0, 5.0]	**<0.001**
Stroke education *N*, (%)		1,064 (70.5)	1,217 (71.2)	186 (70.7)	0.94
**Comorbidity** ***N*****, (%)**					
HF		302 (20.0)	363 (21.2)	108 (41.1)	**<0.001**
CKD		368 (24.4)	434 (25.4)	111 (42.2)	**<0.001**
ESRD		41 (2.7)	49 (2.9)	14 (5.3)	0.055
DM		625 (41.4)	725 (42.40)	158 (60.1)	**<0.001**
HTN		1,054 (69.8)	1,198 (70.1)	183 (69.6)	0.93
Obesity		271 (18.0)	312 (18.3)	64 (24.3)	**0.024**
AF		266 (17.6)	315 (18.4)	82 (31.2)	**<0.001**
Hyperlipidemia		1,306 (71.5)	1,146 (72.4)	160 (65.6)	**0.033**
**HbA1c** ***N*****, (%)**	<5.7	561 (42.1)	492 (42.6)	69 (38.8)	0.067
	5.7–6.4	360 (27.0)	321 (27.8)	39 (21.9)	
	6.5–6.9	92 (6.9)	76 (6.6)	16 (9.0)	
	>6.9	321 (24.0)	267 (23.1)	54 (30.3)	
**NIHSS** ***N*****, (%)**	0	473 (24.8)	431 (26.1)	42 (16.5)	**<0.001**
	1–4	854 (44.8)	749 (45.3)	105 (41.3)	
	5–15	452 (23.7)	370 (22.4)	82 (32.3)	
	16–20	68 (3.6)	58 (3.5)	10 (3.9)	
	>20	59 (3.1)	44 (2.7)	15 (5.9)	
Thrombectomy *N*, (%)		108 (5.5)	91 (5.3)	17 (6.5)	0.54
**Discharge destination** ***N*****, (%)**	Home/home health	1,459 (73.9)	1,299 (76.0)	160 (60.8)	**<0.001**
	Inpatient rehab	290 (14.7)	236 (13.8)	54 (20.6)	
	Skilled nursing facility (SNF)	224 (11.4)	175 (10.2)	49 (18.6)	

AF, atrial fibrillation; CCI, Charlson comorbidity index; CKD, chronic kidney disease; DM, diabetes mellitus; ESRD, end stage renal disease; HF, heart failure; HTN, hypertension; IQR, interquartile range; NIHSS, national institutes of health stroke scale; OR, odds ratio. Bold values indicate statistical significance.

**Table 4:** Multiple logistic regression after model selection for hospital readmission within 90 days.

**Variable**	**OR 95% CI**	***p*-value**
HF	1.51 (1.08–2.11)	**0.015**
CKD	1.28 (0.94–1.75)	0.12
Diabetes	1.50 (1.13–2.01)	**0.006**
AF	1.40 (1.00–1.94)	**0.047**
Increasing Age	1.01 (0.99–1.02)	0.085
Increasing CCI	1.12 (1.03–1.21)	**0.006**
**Discharge destination**		
Inpatient rehab	1.74 (1.22–2.47)	**0.002**
Skilled nursing facility	1.66 (1.13–2.45)	**0.01**
Home/Home health	Reference	

AF, atrial fibrillation; CCI, Charlson comorbidity index; CKD, chronic kidney disease; DM, diabetes mellitus; HF, heart failure; IQR, interquartile range; OR, odds ratio; N/A, Not applicable. Bold values indicate statistical significance.

### 3.3 Outpatient follow-up

There were 424 (21.5%) patients who completed an outpatient stroke clinic follow-up within 30 days of discharge, including 72 patients (3.6%) who completed a telemedicine outpatient visit. Of those, 403 patients were seen in a stroke clinic and 31 in a general neurology clinic. The univariate logistic regression models suggested that the completion of a stroke clinic follow-up within 30 days of discharge was significantly associated with a lower likelihood of 90-day readmission (OR 0.68, 95% CI 0.52–0.90; *p* = 0.006). Patients who completed the first stroke clinic follow-up with a medical doctor compared to those seen by an advanced practitioner had lower odds of an ED revisit within 90 days, though not statistically significant (OR 0.82, 95% CI 0.23–2.91, *p* = 0.77). Similarly, those seen in-person had comparable odds compared to those seen via telemedicine (OR 0.92, 95% CI 0.48–1.75, *p* = 0.80), also not significant.

## 4 Discussion

We found that ED revisits and hospital readmissions within 90 days were associated with patient comorbidities including HF, AF, DM, increasing CCI score, and discharge destination. We also observed that early outpatient follow-up (within 30 days) in an outpatient dedicated stroke clinic was associated with a significant reduction in 90-day readmission rates.

Patients who suffer from AIS frequently have coexisting medical conditions such as HF (15), AF (16), DM (17) and HTN (18), all of which negatively impact patient outcomes, stroke severity, the rate of hospital revisits as well as the cost of care. For instance, patients with uncontrolled HTN at presentation often experience larger infarct volumes and smaller salvageable tissues (19), while those with DM more often experience early neurological deterioration (2) (defined as NIHSS change of ≥4 in the first 72 h). These not only manifest in a higher hospital revisit rate due to worse outcomes, but also in higher hospitalization costs (20). The impact of this is also captured utilizing the CCI scoring system, which estimates the 10-year survival rate based on comorbidity burden (14). One meta-analysis showed that patients with a CCI score >2 had a nearly fourfold increase in the risk of mortality [OR 3.80, 95% CI (1.20–12.01)] (21). Although we excluded patients who died before discharge, our results align with these results, as an increasing CCI score independently increased morbidity burden via unplanned ED and hospital revisits. In another study, it was shown a CCI score of 3 or more was an effective predictor of length of stay and hospital cost (22). These results underscore the necessity of a closer attention to this subset of patients, as recurrent readmissions not only translate clinically into a poor clinical outcome with reduced survival rates, but also contribute to increased healthcare costs (22, 23). Our findings are consistent with prior literature demonstrating a worse clinical outcome as well as hospitalization cost that corresponds to an increasing comorbidity burden (14, 20). Identifying these potential predictors serve as an opportunity to implement targeted interventions aimed at reducing readmission rates for patients with AIS. By recognizing individuals with higher risk, clinicians can plan on a closer outpatient follow-up and provide individualized discharge education to address these predictive factors. These strategies are expected to not only improve patient outcome by reducing recurrent hospital revisit rates, but also lowers healthcare costs.

One study showed that stroke patients with HF and chronic obstructive pulmonary disease who had an outpatient follow-up shortly after their hospital discharge were 21% less likely to be readmitted to the hospital [OR/HR 0.79, 95% CI (0.69–0.91), *p* = 0.001] (23). These outpatient visits serve as an opportunity to complete a comprehensive stroke evaluation, thus ensuring appropriate secondary stroke preventative measures, as well as address co-morbidities that are often associated with AIS. In a comprehensive meta-analysis of 47 randomized controlled trials, two main strategies were highlighted to help reduce hospital readmissions: (1) early interventions facilitating the transition from hospital to home, beginning before discharge, and (2) strategies reinforcing patient empowerment (24). Our study further emphasizes the importance of scheduling patients in the clinic, especially within 30 days and prior to their discharge, in preventing unplanned hospital revisits. Implementation of telemedicine visits to supplement in-person visits also helps in overcoming patient-centered barriers and unexpected cancellations due to weather changes, work commitments, caregiver and family unavailability, the lack of transportation, contagious illnesses or childcare responsibilities -all of which reinforce patient and caregiver empowerment.

While our study offers valuable insights with important clinical implications, several limitations need to be acknowledged. First, its retrospective nature could have introduced bias. Second, only 21.5% had outpatient follow-up visits within 30 days, including only 3.6% via telemedicine during the study period limiting a broader assessment of the impact from telemedicine use. Lastly, this study only captured ED revisits and inpatient readmissions within our healthcare system; while our academic healthcare system is the largest in the state of Georgia, hospital visits to other healthcare facilities were not included and therefore may have been underestimated especially in rural geographic areas.

## 5 Conclusion

Ischemic stroke patients with comorbid conditions including AF, DM, and HF, those with a higher CCI score, and discharge destination to an inpatient rehab or skilled nursing facility are associated with an increased likelihood of 90-day ED revisit or hospital readmission. Efforts to increase outpatient stroke clinic access within 30 days following ischemic stroke discharge may help reduce these 90-day ED revisits and readmissions for acute ischemic stroke patients.

## Data Availability

The raw data supporting the conclusions of this article will be made available by the authors, without undue reservation.

## Supplementary material

Supplementary Figure S1 Area under the curve (ED revisits).

Supplementary Figure S2 Area under the curve (hospital readmissions).
