# Supplementary material for: Predictors of unplanned hospital readmissions and emergency department revisits in patients with acute ischemic stroke
Source: Front Neurol. 2025 Oct 21;16:1683753. doi: 10.3389/fneur.2025.1683753 (PMC12584793; doi:10.3389/fneur.2025.1683753)

**Supplementary material:**

**Figure S1: Area under the curve (AUC) for ED revisits.**

*The point estimate and 95% CI of area under curve (AUC) was 0.67, [0.64, 0.70], respectively.*


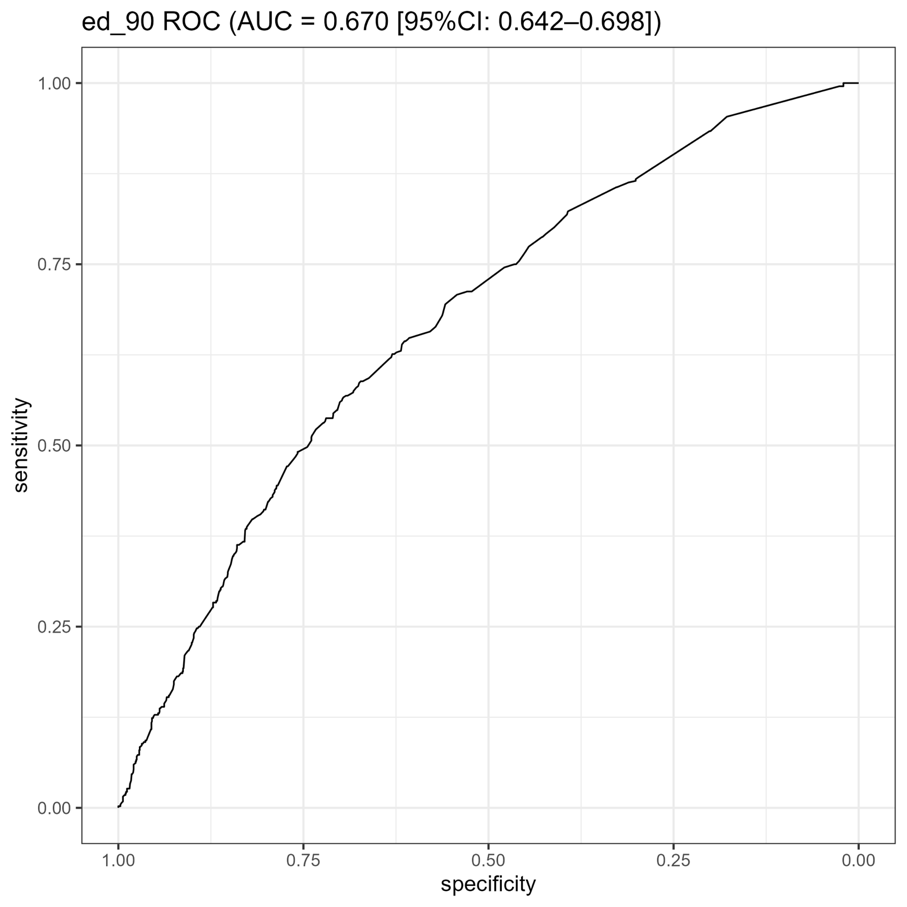


**Figure S2: AUC for Hospital readmission.**

*The point estimate and 95% CI of area under curve (AUC) was 0.70, [0.67, 0.73], respectively.*


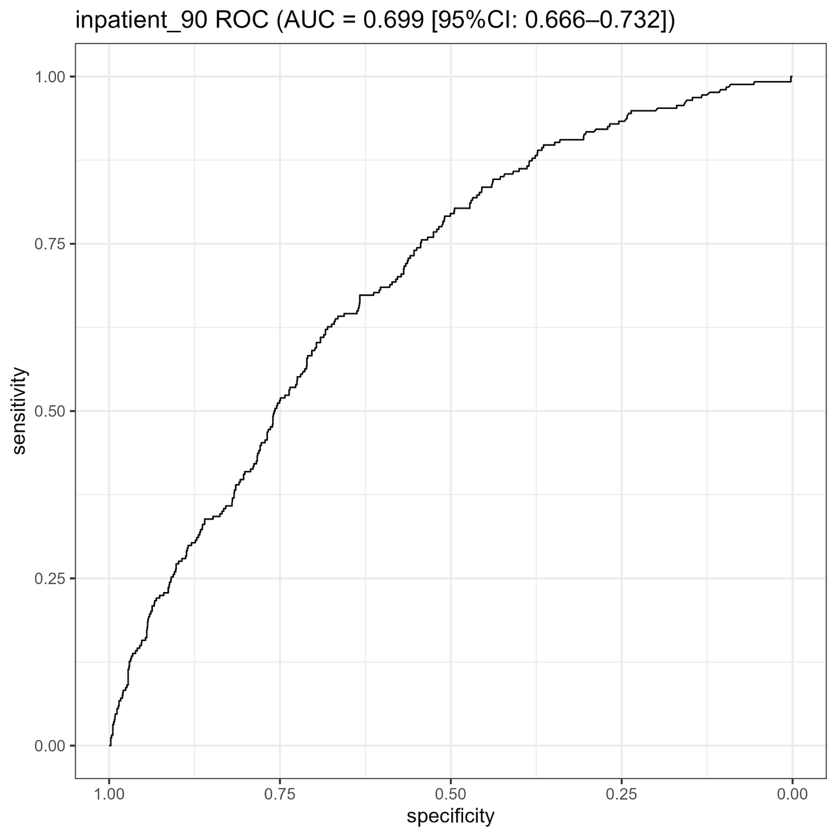

Supplement: Supplementary Figure S1 — Area under the curve (ED revisits). [file Data_Sheet_1.docx]
